# Experiences of Home-Dwelling Older Adults and Their Family Caregivers With Digital Health Services: Qualitative Study

**DOI:** 10.2196/89496

**Published:** 2026-06-11

**Authors:** JiaYi Ruan, CuiXia Guan, QingQing Chen, Ting Yao, Yun Liu, YuXia Zhang, LunFang Xie

**Affiliations:** 1 School of Nursing, Anhui Medical University, No. 81 Meishan Road, Hefei, AnHui, China, 86 13856958216

**Keywords:** older adults, caregiver, digital health service, qualitative study, user experience, health literacy

## Abstract

**Background:**

As the population ages, demand for health services among older adults and caregivers increases. Digital health services help meet this demand. However, acceptance and experiences differ between older adults and caregivers.

**Objective:**

This study aims to explore the specific experiences of home-dwelling older adults and their caregivers with digital health services, identify their willingness to use these services and the factors influencing their adoption, and identify their service needs to inform the subsequent design and optimization of digital health products and services.

**Methods:**

From December 2023 to February 2024, a descriptive qualitative study was conducted. Researchers used purposive and maximum variation sampling to recruit 18 home-dwelling older adults and 17 family caregivers, including 10 matched dyads. Participants came from 7 community health service centers and a tertiary hospital in Hefei, China. Open-ended, semistructured, face-to-face interviews were conducted separately. Data were analyzed using conventional content analysis.

**Results:**

Four main themes emerged. The first were the application characteristics, including usage situations and preferred functions. The second theme described specific service experiences, both positive and negative. The third was the influencing factors. Promoting factors included health needs, user experience, and subjective norms. Obstructive factors differed between groups and included low digital literacy, weak economic foundation, service security concerns, and lack of time. The fourth theme covered suggestions and expectations for improvement. Older adults appreciated the advantages but often had suboptimal experiences due to complex designs and threats to personal dignity. Conversely, caregivers valued efficiency but reported low use, hindered by time constraints and concerns about privacy and safety.

**Conclusions:**

This study reveals a critical gap between the potential and actual use of digital health services. Older adults face barriers with usability and digital literacy. Caregivers struggle to integrate these services and have trust issues. Future optimization requires action at several levels. Government support is needed through policies, authoritative platforms, and subsidies. Service providers should offer age-friendly, personalized design, and continuous support. Social support systems are also essential, such as digital reciprocity from family and peer help. These strategies are critical for enhancing user experience, bridging the digital divide, and enabling both older adults and their caregivers to fully benefit from digital health technologies.

## Introduction

Population aging is a major global challenge that needs immediate action in the 21st century. According to the World Population Prospects 2022 released by the United Nations Department of Economic and Social Affairs (DESA), the proportion of the global population aged 65 years and older is projected to rise significantly from 10% in 2022 to 16% by 2050 [1]. This trend indicates that the global phenomenon of population aging is intensifying continuously. In high-income countries, an aging society has already become a reality. In transitional and low-income countries, the growth of the older adult population is also particularly notable. China, for instance, ranks among the countries at the forefront in terms of both the speed and scale of population aging [2].

With population aging and longer life expectancy, the burden of chronic diseases has grown. As a result, the demand for health management among older adults has increased significantly. Patients with chronic diseases have long-term health management needs. However, due to scarce medical resources, these needs remain unmet. This hinders both health decisions and engagement in self-management [3]. Due to factors such as multimorbidity, low educational attainment, and poor digital health literacy [4], older adults with multiple chronic conditions face more complex and challenging health management barriers [5] and often need to rely on the assistance of caregivers and geriatric nursing staff.

In Europe, general practitioners or family medicine practitioners are considered the cornerstone of primary care [6]. In contrast, health management for older adults in Chinese communities relies more heavily on long-term family caregivers, such as spouses and children [7]. These caregivers may not be professional health care workers, yet they are required to take on tasks including assisting with daily living activities and accessing medical resources. A cross-sectional study on the burden of caregivers for disabled older adults in rural areas found that 77.6% of caregivers had changed their employment status due to caregiving responsibilities [8]. Behind the caregiver role lie unpaid labor, forgone employment opportunities, and economic costs. Chronic diseases such as cardiovascular and cerebrovascular diseases, diabetes, and osteoporosis have a high prevalence among the older population and require long-term management and care. However, as care needs increase, caregivers may experience a decline in their physical health, along with increased social and psychological stress [9,10].

To address the numerous challenges in health management, a growing number of older adults and their caregivers have turned to digital health services. At present, digital health services are widely used as tools for accessing personal health knowledge and conducting self-health management [11]. In low- and middle-income countries, digital health promotion technologies targeting young people have become increasingly prevalent [12]. However, digital technology is not exclusive to young people; a growing number of older adults are breaking stereotypes and beginning to use digital health services to achieve self-health management and access primary care. In China, the implementation forms of digital health services for older adults mainly include health monitoring, health management, daily living assistance, and internet-based diagnosis and treatment [13-15]. In contrast, European countries focus more on the practical applications of digital health services for older adults, with an emphasis on disabled older adults in communities or nursing homes [16-18]. A longitudinal study on the older population indicated that health interventions based on digital health technologies—such as virtual reality technology, wearable devices, telemedicine, artificial intelligence devices, and online health information seeking—have all demonstrated favorable effects in supporting home-based health management for older adults [19].

However, older adults often have limited digital health skills and low health literacy, and the digital divide continues to widen [20]. A scoping review on older adults’ use of digital technologies for health promotion indicated [21] that digital health service technologies will become a primary means for older adults to achieve health goals. However, existing findings have not fully explained the specific application processes of these technologies, and more research is needed to understand the specific experiences of older adults with digital health services [22]. While the unique challenges and pressures faced by family caregivers have attracted considerable attention from researchers, the challenges and experiences they encounter when using digital health services remain poorly understood. Several existing studies suggest that there are specific differences in the perceptions of digital health services between older adults and their caregivers. For example, older adults have a relatively low overall use rate of “Internet+health care,” but a high level of satisfaction with its use, with safety as their primary concern [23]. This indicates that older adults may attach greater importance to safety and privacy protection when choosing digital health services. In contrast, caregivers may focus more on how to effectively use these technologies to improve the quality and efficiency of care [24,25].

The accelerating pace of population aging exerts profound impacts on society, the economy, and public health systems. Addressing these challenges requires comprehensive strategies, including a deep understanding of the health needs of older adults and the support needs of their caregivers, to improve the older adults’ care system and foster social integration and economic development. Therefore, this study aims to conduct an exploratory qualitative study in China to investigate the experiences with digital health services, willingness to use them, and influencing factors from the dual perspective of home-dwelling older adults and their family caregivers. The findings will identify their specific service needs and provide insights for the future design and optimization of digital health products.

## Methods

### Research Design

This study used a descriptive, qualitative design using in-person, semistructured interviews. Through this qualitative research, researchers aimed to understand the specific experiences of older adults and their caregivers with digital health services, identify their usage intentions, influencing factors, and service needs, and provide recommendations to advance the development of digital health services. Furthermore, we adhered to the Standards for Reporting Qualitative Research guidelines [26] to present the findings of this research (see Multimedia Appendix 1).

### Recruitment

The study population included older adults with experience using digital health services and their caregivers. The researchers adopted a purposive sampling method to select research sites in Hefei City, Anhui Province, China, specifically including (1) seven community health service centers: Yaohai District (eastern Hefei), Shushan District (southwestern Hefei), and Baohe District (southern Hefei), which cover the geographical distribution of both old urban areas and emerging development zones and (2) the Geriatric Inpatient Ward of the First Affiliated Hospital of Anhui Medical University. This hospital is the largest public Grade A tertiary general teaching hospital in Anhui Province. The inclusion and exclusion criteria for older adults and caregivers were shown in Table 1. Eligible older adults and caregivers were sought, and participants were briefed on the study objectives and procedures. If they showed interest, they were invited to participate in the study interview. Data saturation was considered to have been achieved when semistructured interviews with successive participants failed to generate any new open-ended codes, categories, or thematic insights on 3 consecutive occasions. The research team then collectively confirmed that no additional meaningful information would be derived from further interviews.

**Table 1.** Inclusion and exclusion criteria for the study population.

Study population	Inclusion criteria	Exclusion criteria
Older adults	Aged ≥60 years Resided in communities in Hefei for ≥6 months Have experiences in using digital health services	Comorbid with other severe diseases Presence of communication disorders
Caregivers	Aged ≥18 years Family members of older adults who assumed primary responsibility for home-based care without receiving remuneration Longest caregiving duration (≥3 months in total) Have experiences in using digital health services Clear consciousness, everyday speech, and the ability to complete the interview	Diagnosis of severe organic diseases or mental illnesses Hired nannies, nursing attendants, or other paid caregivers

### Ethical Considerations

This study has obtained ethical approval from the Biomedical Ethics Committee of Anhui Medical University (Ethical approval number: 83242378). In accordance with the Declaration of Helsinki, the study adheres to the principles of informed consent and privacy protection, and all investigations were conducted with informed consent. All data are strictly confidential and shall be used exclusively for this study. Participants took part in this study voluntarily and did not receive any financial compensation.

### Data Collection

Based on the research objectives, a semistructured interview guide was developed through an extensive review of relevant literature and discussions among research team members. Before the formal interview, we conducted a preinterview with 2 older adults and their caregivers and adjusted the interview questions based on the results. The final interview outline was shown in Multimedia Appendix 1. Prior to the formal interviews, we screened the research participants to ensure that they had experience in using digital health services. At the very beginning of the interview, the research participants were provided with descriptions of the 4 types of digital health services, and then they chose the services they had used in their lives, including health monitoring (Mo), health management (Ma), intelligent life assistance (I), and online health care services (O). Detailed descriptions of the digital health services refer to the outline of the interview in Multimedia Appendix 1. Interviews were conducted in Chinese by 2 researchers (JYR and CXG), who had no prior contact with the participants. Interview data were collected between December 2023 and February 2024 via in-person, semistructured, in-depth interviews, supplemented by field notes. Interviews were arranged at locations convenient to participants and in quiet, undisturbed settings to prevent interruptions. Before each interview, participants’ permission to record audio was obtained.

### Data Analysis

Conventional content analysis guided our analytic approach [27]. Audio recordings were transcribed verbatim immediately after each interview. The textual data were then imported into NVivo (12.0 software; QSR International Pty Ltd). To protect privacy, participants’ real names were replaced with letters. Data analysis, coding, and theme extraction were performed using NVivo 12.0 software. Two researchers (co-first authors JYR and CXG), both of whom had training in qualitative methods, repeatedly read the interview materials (including audio transcripts and field notes) to capture the exact meanings participants intended. JYR repeatedly read the first 4 transcripts, marked all meaning units relevant to the phenomenon, and generated an initial list of codes. Subsequently, JYR and CXG collaborated to develop the codebook. JYR drafted names, definitions, and typical excerpt examples for each initial code, forming the preliminary codebook. The 2 researchers then jointly reviewed the first 4 transcripts, checking the fit between each code and its data source line by line, merging similar codes, refining definitions, and resolving any inconsistencies through discussion. Thereafter, the codebook was applied to subsequent transcripts. When new meanings emerged that could not be captured by existing codes, new codes were added and discussed again for revision. This iterative process continued until no new codes emerged and the codebook was considered saturated and stable.

The final determination of themes was completed by all authors (JYR, CZG, QQC, TY, YL, YXZ, and LFX). After all transcripts had been coded, JYR exported the final list of codes with their definitions and preliminarily clustered them into subcategories and candidate themes based on their shared latent meanings. The research team then held multiple discussions, engaging in repeated debate and revision of the naming, definitions, and boundaries of the themes until consensus was reached. Finally, each theme was assigned a formal name and clear definition, and the most representative participant quotations were extracted as examples.

### Fidelity Monitor

Before initiating the study, researchers received specialized training in interview techniques and conducted pilot interviews, refining the interview guide based on their outcomes. Before formal interviews, recording equipment was tested to ensure optimal functionality and audio quality, and written informed consent was obtained from all participants. Interviews with older adults and their caregivers were independently administered by 2 postgraduate nursing students, with no cross-discussion between the interviewers during data collection. All textual data were anonymized and coded; researchers maintained a strictly neutral stance throughout the analytical process to ensure objective interpretation, and study findings were subjected to rigorous review by the research team to uphold methodological objectivity. In cases of discrepancies in extracted themes, the research team engaged in collective deliberation to reach consensus on final thematic classifications, thereby ensuring the reliability and consistency of the study’s results.

## Results

### Characteristics of Study Participants

The study included 18 older adults with a mean age of 70.17 (SD 6.21) years and 17 caregivers with a mean age of 50.65 (SD 15.37) years. Ten of these participants formed matched older adult and caregiver dyads. Additional demographic characteristics are presented in Multimedia Appendix 1.

### Thematic Findings

The qualitative analysis began with 630 initial codes. Through an iterative process of comparison and consolidation of similar codes, these were refined into 470 distinct codes. These codes were then organized into a hierarchical structure, resulting in 12 subthemes and 4 main themes: (1) usage and preference of services, (2) specific service experiences, (3) influencing factors, and (4) suggestions and expectations. An example of the coding process and the complete coding framework are provided in Multimedia Appendix 1. The similarities and differences in perceptions of digital health services between older adults and caregivers were summarized in Table 2.

**Table 2.** Comparison of perspectives on digital health services between community-dwelling older adults and family caregivers.

Evaluation dimension	Older adults’ perspective	Family caregivers’ perspective	Similarities	Differences (O^a^ and C^b^)
Usage and preference of services	Focus on easy-to-operate, low-cost hardware and basic online services	Usage attached to older adults’ needs; focus on auxiliary monitoring and comprehensive online medical services	Health monitoring and health management services	O: simple hardware and basic functions C: auxiliary operation and full online medical services
Experience	Positive: usefulness, ease of use, health promotion, psychological comfort Negative: low credibility, complex operation, physical discomfort, dignity concerns	Positive: same as older adults Negative: low credibility, complex operation	Value usefulness, ease of use, health promotion, psychological comfort; Face low credibility and complex operation	O: physical discomfort, dignity concerns
Influencing factors	Promoting: health needs, subjective norms, government support Obstructing: low literacy, weak economy, low acceptance of new things	Promoting: health needs, excellent user experience Obstructing: low literacy, cost concerns, low acceptance of new things, insecurity, limited time	Driven by health needs; blocked by low literacy, low acceptance of new things, cost concerns	O: affected by peers/experts/policy; government support C: excellent user experience, limited by time; focus on privacy
Suggestions and expectations	Age-friendly and personalized design; stricter supervision and review; on-site assistance	Age-friendly and personalized design; on-site assistance; family/peer support; authoritative platforms; more publicity	Suggest personalized and age-friendly design, on-site assistance	O: emphasize stricter supervision and review C: emphasize authoritative platforms, publicity, and family/peer support

a O: older adults.

b C: caregivers.

#### Theme 1: Usage and Preference of Services

Online learning enabled older adults to easily acquire health knowledge and skills, thereby enhancing their self-care capacities, while the online shopping function facilitated convenient purchases of health products and services to meet daily needs. Through continuous monitoring via relevant functions, they tracked changes in vital signs like heart rate, blood pressure, and blood sugar, gaining a comprehensive understanding of their health status to detect issues early and prevent complications. The health management feature allowed them to review daily data and obtain personalized advice, reducing hospital visits and enabling effective chronic disease management. Remote diagnosis streamlined health care access, provided tailored services, and allowed them to verify doctors’ qualifications online. Community communication offered a platform for interacting with peers and professionals, sharing experiences, and gaining additional health insights and advice (Textbox 1).

Textbox 1. **Theme 1 and subthemes.**

**Theme 1: Usage and Preference of Services**

**Subtheme 1: Usage situation**

Older adults tended to use health monitoring, health management, and intelligent life assistance services. In contrast, caregivers tended to use health monitoring, health management, and online health care services. A detailed overview of the digital health services used by the older adults and caregivers was presented in Table 3 and Multimedia Appendix 1.

**Subtheme 2: Preferred functions**

Participants reported five preferred functions of digital health services, as follows:

(1) Online learning or shopping

“I took an online traditional Chinese medicine course and really liked the synchronous learning feature, which let me study and practice alongside the video.” [A12, male, 71 years, health management (Ma), online health care services (O)]

“Shopping on the platform is great, and the after-sales service is excellent. We place an order on the platform, and the courier delivers it directly to us.” [A17, female, 72 years, health monitoring (Mo), Ma]

(2) Real-time health monitoring

“With a blood pressure monitor, I can check my blood pressure anytime, anywhere. Seeing a doctor requires going to the hospital, but at home, I can measure it 2 or 3 times a day.” [A13, female, 64 years, Mo]

(3) Health management

“The blood pressure monitor and glucometer are accurate. If my blood pressure is high, it can also remind me to take my medication.” [A5, male, 70 years, Mo]

“This platform also pushes some health information, like how hypertensive patients should control their diet and what they should pay attention to.” [A17, female, 72 years, Mo, Ma]

(4) Remote diagnosis/treatment

“The ‘window’ [to view doctor profiles] is very important; you cannot trust just one doctor's opinion. Although the doctors' introductions on the official account page look similar, you can basically figure it out by looking at a few more... When making an online appointment, you can see the doctor's profile.” [A11, female, 61 years, Ma, O]

(5) Community communication

“He [the staff] shared some precautions and instructions for using the features in the large group chat.” [A17, female, 72 years, Mo, Ma]

“When I come across an article from this official account, I can sometimes share it with others.” [A7, male, 82 years, Ma, intelligent life assistance (I)]

**Table 3.** Digital health services used by older adults and caregivers.

Group	Health monitoring (Mo^a^)					Health management (Ma^b^)					Intelligent life assistance (I^c^)		Online health care services (O^d^)		
	Electronic BP^e^ monitor	Electronic Glucose meter	Smart body fat scale	Wearable activity tracker	Pulse oximeter	Treadmill	Online software and platforms	Health-related short videos	Online purchase of health care products	Professional health websites	Electric wheelchairs	Intelligent cochlear implants	Online appointment and registration	Online health consultations	Online medication purchase
Older adults	✓	✓	✓	✓	✓	✓	✓	✓	✓		✓	✓	✓		
Caregivers	✓	✓	✓	✓			✓	✓	✓	✓			✓	✓	✓

a Mo: health monitoring.

b Ma: health management.

c I: intelligent life assistance.

d O: online health care services.

e BP: blood pressure.

#### Theme 2: Specific Service Experiences

Older adults and caregivers shared highly consistent positive experiences, as both groups acknowledged the value of digital health services in improving medical efficiency, simplifying health management, and alleviating health anxiety, findings that confirmed the adaptability of such services to older adults’ care scenarios. Caregivers’ positive experiences additionally included “reducing care burden,” a benefit closely linked to their role as assistants. In contrast, older adults prioritized the psychological comfort and enhanced life autonomy afforded by the services. Their negative experiences exhibited both commonalities and significant group differences. Both groups encountered issues of low credibility of information and cumbersome operation of certain functions. Older adults, however, experienced unique challenges such as physical discomfort and psychological resistance due to physiological aging and dignity demands, while caregivers focused more on privacy, security risks, and potential costs of service use. Older adults’ negative experiences were the key inducement for their shift from “unable to use” to “unwilling to use.” In contrast, caregivers’ negative feelings directly impacted their willingness to assist older adults in service use (Textbox 2).

Textbox 2. **Theme 2 and subthemes.**

**Theme 2: Specific service experiences**

**Subtheme 1: Positive experience**

Participants reported four key positive experiences with digital health services, as follows:

(1) Perceived usefulness

“With online appointments, you do not have to travel back and forth—that is the most significant convenience. I can book in advance with the specialist who knows my condition. It has many benefits.” [A15, female, 60 years, health monitoring (Mo), health management (Ma), online health care services (O)]

“It is much more convenient. Since we live far away, it would be very troublesome to travel here and find no available appointment slots.” [C10, male, 51 years, Mo]

(2) Perceived ease of use

“It is very simple. You just put your arm in and press a button. The old blood pressure monitors required removing and putting on clothes, which was inconvenient and made our arms sore.” [A2, female, 71 years, Mo, O]

“For example, my grandfather can operate the blood pressure monitor on his own. It saves us both worry and effort, so we will definitely continue using it.” [C2, female, 48 years, daughter of A3, Mo, O]

(3) Promote health management for the older adults

“I bought a treadmill after retiring, partly out of curiosity. It's convenient and novel—I can walk at home without going outside, saving me the need to run outdoors.” [A6, male, 85 years, Ma]

“The health videos are great. Many are from hospital doctors and experts who regularly explain and analyze various diseases. Both the older adults and I can learn a lot from them.” [C2, female, 48 years, daughter of A3, Mo, O]

(4) Recover psychological comfort

“When you go for a walk wearing it [the fitness tracker], you can at least see how many steps you have taken and how far you have gone. It also tells the time. I can check my heart rate when I feel palpitations and my blood oxygen if I feel dizzy. It is definitely helpful, at least I feel less panicked.” [A15, female, 60 years, Mo, Ma, O]

“When older adults feel unwell, they might not tell you, fearing they are a burden, but they worry privately. These health services or short videos can serve as intermediaries, helping with their psychological adjustment.” [C17, female, 33 years, daughter of A11, Mo, Ma]

**Subtheme 2: Negative experience**

(1) Low credibility

“At our age, we care about health, but some online health information is reliable, and some is not... For example, ginger tea, brown sugar, and red dates warm the stomach in winter, but some public accounts claim they have anti-aging effects. That's just exaggerated.” [A15, female, 60 years, Mo, Ma, O]

“I browse on my phone. If something does not seem real, I stop reading and ignore it.” [C4, female, 76 years, spouse of A7, Mo]

(2) Complex operation

“For instance, when a video talks about uric acid, it stops at the key point and requires me to follow a public account to learn more. It's too complicated.” [A1, male, 70 years, Mo, intelligent life assistance (I)]

“The glucometer is also relatively cumbersome to use. It involves a blood draw, so I always help them with the measurement.” [C11, female, 22 years, granddaughter of A4, Ma, O]

(3) Increase physical and mental burden

“We aren't like young people who like wearing earphones. I have ear problems, but when I wear the [cochlear implant], I feel dizzy and my head feels stuffy if I wear both, so I only wear one.” [A7, male, 82 years, Ma, I]

“I have opened those little apps on the phone, but the interfaces pop up everywhere, and you need to click different places. I do not understand it; it is very annoying, so I close it.” [A16, female, 70 years, Mo]

“When my condition flares up, one leg is immobile. The doctor told me to use an electric wheelchair and have a family member push me. However, I think since I can walk with one leg, I still want to walk by myself. Otherwise, I feel I have no dignity.” [A1, male, 70 years, Mo, I]

#### Theme 3: Influencing Factors

The facilitating factors for the use of digital health services were jointly driven by older adults’ internal health needs and external environmental support. Chronic diseases and declining physical function among older adults served as the core internal motivations, while high-quality user experience, subjective norms within social circles, and government policy support were the key external driving forces. Subjective norms exerted a particularly significant impact on older adults, reflecting their susceptibility to guidance from authoritative entities and social circles when accepting new things, whereas caregivers’ willingness to use such services was mostly determined by whether the services could reduce their care burden. The core obstacles differed between the two groups. Older adults faced low digital health literacy, a weak economic foundation, and low acceptance of new things, while caregivers encountered issues such as insufficient time, privacy security concerns, and limited personal digital capabilities. Both groups were affected by inadequate age-friendly service designs; economic costs and service security were common concerns, acting as core external obstacles to the popularization of digital health services, while caregivers’ time constraints were an easily overlooked important influencing factor (Textbox 3).

Textbox 3. **Theme 3 and subtheme.**

**Theme 3: Influencing Factors**

**Subtheme 1: Promoting factors**

Participants reported four driving factors affecting usage, as follows:

(1) Health needs

“I have diabetes and hypertension myself, so I need to keep track of my blood sugar and blood pressure levels.” [A4, male, 70 years, health monitoring (Mo)]

“My hearing is very poor, which makes communication difficult. He [the older adult] can't hear clearly at all when others speak.” [C4, female, 76 years, spouse of A7, Mo]

(2) Excellent user experience

“It is definitely the effectiveness [of the online health platform]. It has practical benefits for the older adult and is helpful for me, too.” [C7, female, 52 years, daughter of A17, health management (Ma), intelligent life assistance (I)]

“The advantage is that it's simple and easy to operate. We will certainly continue using it.” [C2, female, 48 years, daughter of A3, Mo, online health care services (O)]

(3) Subjective norms

“My social circle shares information about nutrition and health, and there's a sense that we older adults should keep up with the times and learn to use some smart health devices.” [A9, male, 65 years, Ma]

“When browsing Douyin or WeChat, I come across health-related videos, such as those from wellness experts or doctors doing science popularization.” [A7, male, 82 years, Ma, I]

(4) Government support

“The government is now strongly promoting the ‘Big Health’ initiative. Following government policy is undoubtedly the right thing to do, and it is also suitable for us older people.” [A17, female, 72 years, Mo, Ma]

**Subtheme 2: Obstructive factors**

(1) Low level of digital health literacy

“We don't have a medical background, so it’s hard to know what's true or false. We also don’t understand how to use high-tech products.” [A12, male, 71 years, Ma, O]

“Smartphone technology is advancing too fast. It is tough for older minds to adapt. Our [referring to caregivers] education and cultural level have not reached that high standard either.” [C16, male, 47 years, son of A10, Mo]

(2) Weak economic foundation

“I’m from the countryside. My children have heavy burdens; sometimes they can't even take care of themselves.” [A10, female, 70 years, Mo]

“If we were to continue using it, the first thing [we consider] would definitely be the price.” [C14, male, 50 years, son of A8, Mo, O]

(3) Lack of awareness of some digital health services

“I personally believe that seeing a doctor still requires going to the hospital. We only feel reassured after hearing a professional doctor's advice.” [A6, male, 85 years, Ma]

“Without actually using this thing [digital health device], you don't know where its benefits lie.” [C14, male, 50 years, son of A8, Mo, O]

(4) Low service security

“With online consultations, we always feel somewhat distrustful of the platform. We’re afraid that leaving personal information could lead to fraud.” [C2, female, 48 years, daughter of A3, Mo, O]

(5) Little free time

“I am quite busy with work now and have no time to watch [health videos, etc.]. I spend my evenings accompanying the two older adults, especially my dad, so sometimes I don't have much time to deal with other things [like exploring other digital health services].” [C7, female, 52 years, daughter of A17, Ma, I]

#### Theme 4: Suggestions and Expectations

Participants advocated for simplified operation procedures, age-adapted interface designs, and personalized service configurations to enhance accessibility and meet older adults’ diverse needs, while older adults emphasized the necessity of strengthened supervision and review mechanisms to protect data privacy, property security, and standardized advertising content. To address technical and operational barriers, older adults expected regular on-site assistance and professional in-home guidance, whereas caregivers highlighted the significance of community support and professional services. Caregivers acknowledged the positive role of family members, peers, and social circles in facilitating older adults’ acquisition of knowledge and acceptance of digital health services through technical and emotional support. They also proposed establishing official hospital-operated health accounts with physicians as core health information disseminators, whose institutional endorsement could significantly improve information trustworthiness. Additionally, caregivers stressed that insufficient publicity and low public recognition, particularly among older adults, hindered service acceptance, and multichannel promotion was deemed effective to expand coverage, raise awareness, and build public trust (Textbox 4).

Textbox 4. **Theme 4 and subthemes.**

**Theme 4: Suggestions and Expectations**

**Subtheme 1: Improve the age-friendly and personalized design of the services**

“It’s worth continuing to promote these services. I suggest adding more options in form to cater to the personalized needs of older adults.” [A4, male, 70 years, health monitoring (Mo)]

“Text is definitely better for us because our reactions are slow, and we can ponder the text. Audio finishes playing and is easily forgotten.” [A11, female, 61 years, health management (Ma), online health care services (O)]

“The online part should be made as simple as possible, so it’s easier for older people like us, or even older ones, to operate. Using audio instead of text would be very convenient for illiterate seniors.” [C2, female, 48 years, daughter of A3, Mo, O]

**Subtheme 2: Strengthen the intensity of supervision and review**

“(Some advertisers) use celebrity endorsements to deceive people. If we believe them, we pay directly by scanning a code on the platform, and end up losing our money. This needs proper regulation.” [A18, female, 66 years, Mo]

**Subtheme 3: Offer on-site assistance**

“It would be great if there were professionals, for example, coming to our home once a month to calibrate the devices.” [A18, female, 66 years, Mo]

“It would be better if the community had professionals. If experts (like family doctors) could provide in-home guidance or regularly help us check things, they [the older adults] would certainly find it convenient and wouldn’t need to go out.” [C8, female, 42 years, daughter of A18, Ma]

**Subtheme 4: Encourage digital feedback and peer support**

“If an older adult doesn't live with their children, exposure should start within their own social circle. If they live with their children, the children should take the lead in introducing the services. Guidance and information passed on by children are certainly more readily accepted by older adults.” [C1, male, 37 years, son of A2, Mo, intelligent life assistance (I)]

**Subtheme 5: Establish an authoritative platform**

“Now I see many videos are made by doctors’ personal private accounts. Could a dedicated hospital account be created, where doctors from various departments regularly explain medical knowledge about different diseases? Using the hospital as the main body would be better—it's more comprehensive and, secondly, more credible. [C2, female, 48 years, daughter of A3, Mo, O]

**Subtheme 6: Increase the level of publicity**

“Many people don't trust online health product platforms; my mother didn’t believe in it at first. Therefore, it’s necessary to increase publicity efforts and enhance credibility among older adults.” [C7, Female, 52 years, daughter of A17, Ma, I]

“It's definitely worthwhile. This thing [digital, health services] must be popularized.” [C1, male, 37 years, son of A2, Mo, I]

## Discussion

### Principal Findings

This study identified four key findings regarding digital health service experiences among home-dwelling older adults and their family caregivers. First, both groups primarily used health monitoring and basic health management functions but showed distinct preferences for service types. Second, they shared positive experiences of improved convenience and health support, while older adults uniquely reported dignity-related distress and physical discomfort, and caregivers emphasized privacy and safety concerns. Third, usage was driven by health needs and social influences but blocked by low digital literacy, economic constraints, and time shortages. Fourth, both groups prioritized age-friendly design, authoritative platforms, on-site guidance, and family or peer support to optimize future services.

### Usage and Preference of Services

In general, the types of services frequently used by older adults include health monitoring, health management, artificial intelligence–powered living assistance, and Internet-based medical services, which echo the categories of customer-system interaction, decision support, and service delivery in the World Health Organization (WHO) classification of digital health interventions [28]. In addition, regarding health monitoring and management, older adults showed high acceptance and demand. Older adults tend to use functions that support their autonomy in daily life and show a strong preference for low-cost or free services, such as using an electronic blood pressure monitor and an electronic glucose meter, and accessing online health information. Although digital technology has shown great potential for improving health services for older adults [29], the penetration rate of digital technology in transitional and low-income countries is low, older adults’ purchasing power is limited, and their health literacy is low. Moreover, older adults in rural and remote areas often face challenges, including inadequate infrastructure and limited access to technology [30]. Therefore, it is reluctant to try services with high technical access requirements, complex operational processes, and high use costs, such as hip protective airbags, smart homes, and body sensors.

### Service Experience

#### Common Experience

Both older adults and their caregivers can benefit from digital health services, but they also have the same negative experiences. First, both recognized the value of digital health services in improving the efficiency of medical treatment, simplifying health management, and providing psychological comfort. At the same time, it faces the dual challenges of low service reliability and high operational complexity. At present, the promotion of digital health services mainly relies on traditional media, online media, and medical institutions [31]. The COVID-19 pandemic fast-tracked the digital transformation of health care [32]. Some digital health service tools can meet people’s daily health management needs, improve their understanding of their own health status, thereby relieving the tension and anxiety caused by uncertainty about their bodies, reduce unnecessary medical visits, and reduce the burden on the medical system [19]. However, some older adults who are not familiar with modern technology products often feel strange and uneasy when using smartphones, tablets, and other devices. Complex operations and a large volume of health information that is difficult to distinguish between authenticity and inaccuracy will cause older adults to experience technical anxiety and worry about economic losses from accidental contact [33]. Family caregivers play an important role in the lives of older adults. They not only provide daily care but also need to assist them in collecting health information and using communication technology to ensure the quality of life of older adults [34]. Therefore, caregivers’ experience of use is also significantly influenced by older users’ experience, and they tend to be more concerned about the risk of privacy leakage among older people. This may be because they expect that digital health services can adapt to the abilities and needs of older adults, save physical and time costs, and reduce their own care burden [35].

#### Differentiated Experience

In addition, there was a significant difference in the focus of experience between older adult users and caregivers. For many older adults, the Internet breaks down the limitations of physical space, allowing them to participate in social interactions and effectively relieving loneliness and enhancing social belonging [36]. By mastering new technology, older people often gain a sense of control. This increased ability translates directly into a more positive mindset [37]. In contrast, family caregivers affirmed the advantages of digital services in convenience, information access, and remote monitoring. They regarded it as a tool to reduce the burden of care and optimize the efficiency of health management. At the same time, the experience of older people is deeply affected by their physical decline, insufficient digital literacy, and psychological feelings. The impaired vision, hearing, and learning ability make them prone to frustration in the face of pop-up windows and multistep operations, which damages their autonomy and dignity. This is consistent with the study of Moonika et al [38]; when older adults use digital health services, they will feel the loss of control and the increased sense of dependence on others, and this sense of dependence will damage their personal dignity, directly causing some older adults to develop from “cannot use” to “unwilling to use” and actively avoid digital health services. It should be noted that compared with other caregivers, older adults’ spousal caregivers have poor health status [39], lack necessary digital health knowledge and digital skills and ability to solve mental disorders of the older adults [40], and may not be able to effectively help the older adults access and use digital health services, thus limiting the effective use of digital health technology by the older adults.

Therefore, future digital health services must adopt a differentiated and collaborative strategy to effectively integrate into the lives of home-dwelling older adults and their caregivers. On the one hand, it is necessary to systematically lower the usage threshold and reduce the trust cost for older adults by simplifying the interface, making the operation process user-friendly, and ensuring the authority of information sources. On the other hand, digital skills support training should be provided to different types of caregivers to varying degrees, leveraging the family collaboration function and enhancing the collective force of family health management through technological convenience.

### Influencing Factors

The continuous innovation and widespread adoption of digital health technologies have led both older adults and their caregivers to recognize the advantages of digital health services. Research indicated that the fulfillment of health needs and positive user experiences facilitate the willingness of older adults and caregivers to adopt digital health services, which is consistent with the Technology Acceptance Model [41], in which perceived usefulness and perceived ease of use are key influencing factors. In addition, subjective norms, such as encouragement from family members, peers, and health experts and the sharing of positive usage experiences, serve as important references for older adults. This finding aligns with the Technology Acceptance and Use Unified Theory (UTAUT), which emphasizes social influence as a critical factor in technology adoption [42]. Furthermore, older adults demonstrated positive attitudes toward government-supported digital health services [43], which is consistent with the concept of facilitating conditions in UTAUT, reflecting that policy-level promotion can enhance older adults’ awareness and acceptance of digital health services.

Nevertheless, they continue to face multiple barriers in actual use. Older adults generally have low digital health literacy, making it difficult for them to adapt to rapidly evolving internet-based health technologies [22]. The study found that they exhibited avoidance and rejection attitudes toward some digital health services and were more inclined to trust the diagnoses, treatment opinions, and effects of offline medical services. This preference reflects a tendency to adhere to familiar practices even when new alternatives are available. In addition, the financial burden associated with purchasing digital devices or accessing paid digital health services remains a salient challenge for both low-income older adults and caregivers [34].

Caregivers also face barriers related to their roles. Lack of time is a major constraint, particularly for younger adult caregivers, who struggle to balance work, caregiving responsibilities, and personal life, leaving them little time to assist older adults with digital health resources [44]. Although these caregivers recognize the potential benefits of such services, they find it difficult to actively use them, creating a gap between intention and behavior. Older spousal caregivers encounter technical difficulties similar to those faced by older adults. In addition, caregivers expressed heightened concerns regarding safety and privacy, particularly regarding the security of online platforms and the risk of misuse of personal information. They generally perceive older adults as being in a passive position in the digital society and worry about the safety of older adults using digital health services independently, tending to restrict their use [45].

The Technology Acceptance Model and UTAUT models emphasized rational cognitive factors and ignored the influence of additional factors on users’ behavioral intentions, such as time, cost, safety, emotions, and whether they possess the necessary skills to use digital services. Therefore, these classic models were insufficient to explain older adults’ adoption behaviors of digital health services. Some studies [46,47] had attempted to extend these models by adding new variables or integrating with other theoretical frameworks to make them more applicable to older populations in digital health. Future research should extend these classic models by integrating insights from qualitative findings, constructing a theoretical framework that is more tailored to older adults for digital health services.

### Suggestions and Expectations

Based on feedback from older adults and their caregivers, this study proposes strategies to optimize the adoption of digital health services from 3 dimensions, namely government, service providers, and social support. The government should rely on grassroots institutions such as communities to establish an integrated digital health literacy education, training, and promotion system that covers the continuum from home to community and from offline to online [48], enhancing older adults’ comprehension of and experience with digital health technologies, thereby facilitating the widespread adoption of digital health services among this population. Additionally, it is recommended that economic incentives be implemented to reduce usage costs—for example, providing subsidies for digital devices and including relevant expenses in medical insurance reimbursement—to alleviate financial barriers for older adults and low-income groups. For instance, in Nanjing, China, the government provides digital devices, such as smart bracelets, to older adults as a welfare benefit [49,50]. Older adults can access services either through automatic feedback from the devices or through active calls, improving service accessibility and acceptability. Simultaneously, the government should strengthen platform supervision, improve mechanisms for digital privacy protection, and establish authoritative information communication channels jointly led by multiple formal medical institutions to enhance public trust in and adoption of such services. Digital health service providers emphasize the need for age-appropriate, personalized design centered on older adults’ needs, with a focus on simplifying operational processes, optimizing user interface interactions, and accounting for age-related physical and cognitive characteristics [51]. Design should be grounded in older users’ actual usage habits to lower technological barriers. Previous research indicates that interface adaptability and user-centered design can significantly enhance older adults’ engagement [52]. For example, older adults have suggested that platforms should offer multiple input and output methods (eg, text and audio) to meet the needs of users with varying educational levels and processing speeds. In terms of after-sales support, it is recommended to establish user profiles, conduct regular follow-ups on usage experiences, and set up after-sales service stations in communities, pharmacies, and other locations to provide continuous support. Additionally, service providers should formulate reasonable pricing in line with government guidance, offer discounts or medical insurance support for specific older adult groups, and balance social responsibility with economic benefits. Social support plays a crucial role in helping older adults improve their digital health literacy. “Digital reciprocity”—where adult child caregivers support older adults in learning and using digital health services—is recognized as the most direct and practical approach. Face-to-face guidance from adult child caregivers can encourage older adults to adopt an open attitude toward digital technology, gradually guiding them to accept and actively learn health knowledge, thereby enabling older adults to receive both technical support and positive emotional incentives [53]. If adult child caregivers are unable to assist due to time constraints or if older adult spousal caregivers themselves face barriers to using digital technology, social forces such as peer groups, community organizations, or young volunteers can be leveraged to establish a “community school” model, forming an extra-familial reciprocity structure [41]. This mechanism can help older adults overcome technology anxiety, better perceive the value of digital health services, and enhance their intention to use and self-efficacy.

### Limitations and Future Directions

This study had several limitations. First, regarding the sample selection, the study was confined to Hefei, China, which may limit the generalizability of the findings to regions with different health care and digital infrastructure contexts. Second, to respect participants’ willingness and to capture diverse attitudes and experiences, older adults and their caregivers were not fully paired: only 10 dyads were matched, while the remaining participants were recruited independently. This partial pairing reduced the strength of dyadic-level interpretations, although separate analyses still provided valuable insights into each group’s experiences and needs. Third, all participants had prior experience with digital health services, which may have introduced a positive bias toward perceived benefits and facilitators. Future research should adopt large-scale, multicenter sampling strategies, prioritize the recruitment of fully paired older adult and caregiver dyads, and include participants with varying levels of digital health experience to enhance representativeness.

Beyond sample-related improvements, future studies should also investigate the heterogeneity of digital health services themselves. Specifically, within the same population, older adults’ experiences, acceptance, and barriers may differ significantly across various types of digital health services. Exploring these intraindividual differences would help identify which service characteristics are most conducive to adoption and sustained use among older adults. Such insights are critical for tailoring service design and implementation strategies to diverse user needs and contexts.

### Conclusion

This study analyzed the usage of digital health services from the dual perspectives of older adults and their caregivers. Although all participants could benefit from digital health services, their willingness to use digital health technologies remained low due to various barriers, including low digital health literacy, weak economic foundations, and inadequate service security. Therefore, it is suggested that future promotion and optimization of digital health services be advanced collaboratively across three dimensions: government guidance, service provider optimization, and the construction of social support systems. This integrated approach aims to improve the user experience for older adults and caregivers, facilitate sustained adoption of these services, and ultimately enable older adults to benefit from digital health technologies.

## Supplementary material

10.2196/89496 Multimedia Appendix 1 Appendix reporting standards, interview guides, participant demographics, and coding framework.

## Abbreviations

DESA: Department of Economic and Social Affairs
UTAUT: Unified Theory of Acceptance and Use of Technology
WHO: World Health Organization
