# Supplementary material for: Experiences of Home-Dwelling Older Adults and Their Family Caregivers With Digital Health Services: Qualitative Study
Source: J Med Internet Res. 2026 Jun 11;28:e89496. doi: 10.2196/89496 (PMC13255930; doi:10.2196/89496)
Supplement: Multimedia Appendix 1 [file jmir-v28-e89496-s001.docx]

**Appendix 1**

**Standards for Reporting Qualitative Research (SRQR)***

<http://www.equator-network.org/reporting-guidelines/srqr/>

**Page/line no(s).**

**Title and abstract**

| **Title** - Concise description of the nature and topic of the study, Identifying the  study as qualitative or indicating the approach (e.g., ethnography, grounded  theory) or data collection methods (e.g., interview, focus group) is recommended | P1, lines 1~2 |
| --- | --- |
| **Abstract** - Summary of key elements of the study using the abstract format of the intended publication; typically includes background, purpose, methods, results, and conclusions. | P1~P2, lines 3~41 |

**Introduction**

| **Problem formulation** - Description and significance of the problem/phenomenon studied; review of relevant theory and empirical work; problem statement | P2~P4, lines 46~117 |
| --- | --- |
| **Purpose or research questio**n - Purpose of the study and specific objectives or questions | P4~P5, lines 118~127 |

**Methods**

| **Qualitative approach and research paradigm** - Qualitative approach (e.g.,  ethnography, grounded theory, case study, phenomenology, narrative research) and guiding theory if appropriate; identifying the research paradigm (e.g.,  postpositivist, constructivist/ interpretivist) is also recommended; rationale** | P5, lines 131~137 |
| --- | --- |
| **Researcher characteristics and reflexivity** - Researchers’ characteristics that may influence the research, including personal attributes, qualifications/experience, relationship with participants, assumptions, and/or presuppositions; potential or actual interaction between researchers’ characteristics and the research questions, approach, methods, results, and/or transferability | P8, lines 207~219 |
| **Context** - Setting/site and salient contextual factors; rationale** | P5, lines 139~147 |
| **Sampling strategy** - How and why research participants, documents, or events  were selected; criteria for deciding when no further sampling was necessary (e.g., sampling saturation); rationale** | P5, lines 147~150 |
| **Ethical issues pertaining to human subjects** - Documentation of approval by an appropriate ethics review board and participant consent, or explanation for lack thereof; other confidentiality and data security issues | P6, lines 157~163 |
| **Data collection methods** - Types of data collected; details of data collection  procedures including (as appropriate) start and stop dates of data collection and analysis, iterative process, triangulation of sources/methods, and modification of procedures in response to evolving study findings; rationale** | P6-P7, lines 164-192 |
| **Data collection instruments and technologies** - Description of instruments (e.g., interview guides, questionnaires) and devices (e.g., audio recorders) used for data collection; if/how the instrument(s) changed over the course of the study | P6-P7, lines 164~192 |
| **Units of study** - Number and relevant characteristics of participants, documents, or events included in the study; level of participation (could be reported in results) | P8, lines 222~225 |
| **Data processing** - Methods for processing data prior to and during analysis,  including transcription, data entry, data management and security, verification of data integrity, data coding, and anonymization/de-identification of excerpts | P7, lines 194~197 |
| **Data analysis** - Process by which inferences, themes, etc., were identified and  developed, including the researchers involved in data analysis; usually references a specific paradigm or approach; rationale** | P7~P8, lines 197~205 |
| **Techniques to enhance trustworthiness** - Techniques to enhance trustworthiness and credibility of data analysis (e.g., member checking, audit trail, triangulation); rationale** | P8, lines 206~219 |

| **Synthesis and interpretation** - Main findings (e.g., interpretations, inferences, and themes); might include development of a theory or model, or integration with  prior research or theory | P9~P24, lines 235~664 |
| --- | --- |
| **Links to empirical data** - Evidence (e.g., quotes, field notes, text excerpts, photographs) to substantiate analytic findings | P9~P24, lines 235~664 |

**Results/findings**

**Discussion**

| **Integration with prior work, implications, transferability, and contribution(s) to the field -** Short summary of main findings; explanation of how findings and conclusions connect to, support, elaborate on, or challenge conclusions of earlier scholarship; discussion of scope of application/ generalizability; identification of unique contribution(s) to scholarship in a discipline or field | P25~P30, lines 666~789 |
| --- | --- |
| **Limitations** - Trustworthiness and limitations of findings | P30~P31, lines 838~852 |

**Other**

| **Conflicts of interest** - Potential sources of influence or perceived influence on study conduct and conclusions; how these were managed | P33, lines 907 |
| --- | --- |
| **Funding** - Sources of funding and other support; role of funders in data collection, interpretation, and reporting | P32, lines 875~882 |

| *The authors created the SRQR by searching the literature to identify guidelines, reporting standards, and critical appraisal criteria for qualitative research; reviewing the reference  lists of retrieved sources; and contacting experts to gain feedback. The SRQR aims to  improve the transparency of all aspects of qualitative research by providing clear standards for reporting qualitative research. **The rationale should briefly discuss the justification for choosing that theory, approach, method, or technique rather than other options available, the assumptions and limitations implicit in those choices, and how those choices influence study conclusions and  transferability. As appropriate, the rationale for several items might be discussed together. |
| --- |

Reference:

O'Brien BC, Harris IB, Beckman TJ, Reed DA, Cook DA.Standards for reporting qualitative research: a synthesis of recommendations. Academic Medicine, Vol. 89, No. 9 / Sept 2014

DOI: 10.1097/ACM.00000000000003

**Appendix 2**

**Outline of the interview**

**Guide**: The researchers introduced the specific meanings of four types of digital health services to the respondents, enabling them to clearly identify which category of digital health services they were using.

**①Health monitoring (Mo):** A device that helps monitor blood pressure, blood glucose, and other physical indicators. Such as intelligent tunnel-type blood pressure monitors, electronic blood pressure monitors, electronic blood glucose meters, smart body fat scales, pulse oximeters, wearable activity trackers (e.g., wrist-worn devices that could measure sleep, blood oxygen, and heart rate), and smart home monitoring devices.

**②Health management (Ma):** A platform, software, or device that helps with health management and provides health-related information. Such as automatic physical examination machines, treadmills, training devices, online health management software and platforms (e.g., sports health apps, health information websites, Toutiao, Baidu, WeChat official accounts, WeChat steps, Weibo), health related short videos (e.g., TikTok), online purchase of health care products, and professional health websites (e.g., DingXiang Doctor, Boohee Health, Xunfei Xiaoyi).

**③Intelligent life assistance (I):** Artificial intelligence auxiliary equipment in daily life. Such as intelligent home, intelligent wheelchair, intelligent walking aid, and intelligent hearing aid.

**④Online healthcare services (O):** Relying on Internet platforms to seek medical treatment and purchase medical supplies. Such as online appointment registration, online health consultation, online hospital, online chronic disease follow-up, appointment diagnosis and treatment, intelligent follow-up, mobile intelligent on-site diagnosis and treatment, and cloud pharmacy distribution (e.g., online medication purchase).

If necessary, pictures and videos were combined to help the interviewees understand.

The interview outline for the older adults and caregivers was as follows:

**Ⅰ Outline of interviews with older adults**

1. What digital health services had you used?
2. What were your reasons for starting to try this/these health services? (If two or more types of digital health services were involved, the following questions were asked separately.)
3. How did you feel when you used it? If so, to what extent? In what ways?
4. Which function/part of the digital health service product did you often use, and why did you like to use this function/part?
5. What were your reasons for continuing to use this/these health services?
6. What were the main difficulties you faced when using health services? How did you solve them?
7. What other digital health services had you heard about? Why were they not used?
8. Were these health services worth promoting? What areas needed to be improved? (Suggestion were made.)

**Ⅱ Outline of family caregiver interviews**

1. What digital health services had you used to better care for the older adult?
2. What motivated you to continue using it/them?
3. What prevented you from continuing to use it/them?
4. Did you continue to use it/them?
5. What digital health services had the older adult in your family used themselves?
6. Why did older adult in your family want to use digital health services?
7. How did you feel about the older adult in your family using digital health services?
8. What factors did you think encouraged older people to use digital health services?
9. What did you think were the barriers that hindered older people from using digital health services?
10. Would you recommend digital health services for older adults in the future? Why?
11. What were your suggestions for the future development of digital health services for the older adult?

**Appendix 3**

**Table 1.** Demographic characteristics of Older Adult Participants and their use of digital health services

| **Participant ID** | **Age (Years)** | **Sex** | **Marital Status** | **Educational Level** | **Comorbidities** | **Primary Caregiver** | **Types of**  **service** | **Name of service** |
| --- | --- | --- | --- | --- | --- | --- | --- | --- |
| A1 | 70 | Male | Married | Primary School | Hypertension, Arthritis | Spouse | Mo, I | Electronic BP monitor, Electric wheelchairs |
| A2* | 71 | Female | Married | Illiterate | Hypertension, Diabetes | Son | Mo, O | Electronic BP monitor, Electronic glucose Meter, Online health consultations |
| A3* | 72 | Male | Married | Junior High School | No chronic conditions | None | Mo | Electronic BP monitor,  Smart body fat scale |
| A4* | 70 | Male | Married | Junior High School | Hypertension, Diabetes | Spouse | Mo | Electronic BP monitor,  Electronic glucose Meter |
| A5 | 70 | Male | Married | Primary School | Parkinson's Disease | Spouse | Mo | Electronic BP monitor,  Electronic glucose Meter |
| A6 | 85 | Male | Married | Technical Secondary School | Hypertension | Spouse | Ma | Treadmill |
| A7* | 82 | Male | Married | Technical Secondary School | Coronary Heart Disease | Spouse | Ma, I | Intelligent cochlear implants, Health related short videos |
| A8* | 77 | Female | Married | Primary School | Cerebral Infarction, Cardiac Insufficiency, Pulmonary Insufficiency | Son | Ma | Online software and platforms |
| **Participant ID** | **Age (Years)** | **Sex** | **Marital Status** | **Educational Level** | **Comorbidities** | **Primary Caregiver** | **Types of**  **service** | **Name of service** |
| A9* | 65 | Male | Married | Junior College | Hypertension | Spouse | Ma | Professional health websites |
| A10* | 70 | Female | Married | Primary School | Hypertension, Diabetes | Son | Mo | Electronic BP monitor |
| A11* | 61 | Female | Married | Primary School | Hypertension | Daughter | Ma, O | Health related short videos, Online appointment and registration |
| A12 | 71 | Male | Married | Junior College | Rheumatism, Lumbar Disc Herniation | Spouse | Ma, O | Online health consultations,  Health related short videos |
| A13 | 64 | Female | Married | Illiterate | Myocardial Ischemia | Spouse | Mo | Electronic BP monitor |
| A14 | 67 | Male | Married | Primary School | Diabetes, Hyperlipidemia | Spouse | Mo, O | Electronic glucose Meter, Online appointment and registration,  Online medication purchase |
| A15 | 60 | Female | Married | Senior High School | Rheumatoid Arthritis | Spouse | Mo, Ma, O | wearable activity tracker, Online appointment and registration, Treadmill |
| A16 | 70 | Female | Married | Primary School | Hypertension, Cerebral Infarction, Sciatica | Spouse | Mo | Electronic BP monitor |
| A17* | 72 | Female | Married | Bachelor's Degree | Hypertension | Daughter | Mo, Ma | Online purchase of health care products, Electronic BP monitor |
| A18* | 66 | Female | Married | Junior High School | Hypertension | Daughter | Mo | Pulse oximeter, Electronic BP monitor |

Note: An asterisk indicates that the participant's corresponding caregiver was also interviewed.

**Table 2.** Demographic Characteristics of Caregiver Participants and their use of digital health services

| **Caregiver ID** | **Age (Years)** | **Sex** | **Marital Status** | **Educational Level** | **Employment Status** | **Duration of Care** | **Relationship to Older Adult** | **Type of service** | **Name of service** |
| --- | --- | --- | --- | --- | --- | --- | --- | --- | --- |
| C1* | 37 | Male | Married | Junior College | Self-Media Entrepreneur | 10 years | Son | Mo, I | Electronic BP monitor, Electric wheelchairs |
| C2* | 48 | Female | Married | Junior High School | Unemployed | 1 year, 7 months | Daughter | Mo, O | Electronic BP monitor, Electronic glucose Meter, Online health consultations |
| C3 | 48 | Male | Married | Bachelor's Degree | Manager at a Private Medical Check-up Centre | Cohabiting | Son | Mo | Electronic BP monitor, Smart body fat scale |
| C4* | 76 | Female | Married | Junior College | Unemployed | Cohabiting | Spouse | Mo | Electronic BP monitor, Electronic glucose Meter |
| C5 | 32 | Male | Married | Bachelor's Degree | Software Engineer | Cohabiting | Son | Mo | Electronic BP monitor, Electronic glucose Meter |
| C6 | 50 | Male | Married | Senior High School | Community Worker | Cohabiting | Son | Ma | Treadmill |
| C7* | 52 | Female | Married | Senior High School | Accountant | 2 years, 3 months | Daughter | Ma, I | Intelligent cochlear implants, Health related short videos |
| C8* | 42 | Female | Married | Junior College | Unemployed | Cohabiting | Daughter | Ma | Online software and platforms |
| **Caregiver ID** | **Age (Years)** | **Sex** | **Marital Status** | **Educational Level** | **Employment Status** | **Duration of Care** | **Relationship to Older Adult** | **Type of service** | **Name of service** |
| C9 | 58 | Male | Married | Bachelor's Degree | Civil Servant | 1 years | Son | Ma | Professional health websites |
| C10 | 51 | Male | Married | Junior High School | Workshop Worker | Cohabiting | Son | Mo | Electronic BP monitor |
| C11* | 22 | Female | Unmarried | Postgraduate | Student | 1 years | Granddaughter | Ma, O | Health related short videos, Online appointment and registration |
| C12 | 70 | Male | Married | Primary School | Unemployed | Cohabiting | Spouse | Ma, O | Online health consultations, Health related short videos |
| C13 | 83 | Male | Married | Bachelor's Degree | Unemployed | Cohabiting | Spouse | Mo | Electronic BP monitor |
| C14* | 50 | Male | Unmarried | Junior College | Unemployed | 10 years | Son | Mo, O | Electronic glucose Meter, Online appointment and registration, Online medication purchase |
| C15* | 62 | Female | Married | Junior High School | Unemployed | Cohabiting | Spouse | Mo, Ma, O | wearable activity tracker, Online appointment and registration, Treadmill |
| C16* | 47 | Male | Married | Junior High School | Community Worker | 4 years | Son | Mo | Electronic BP monitor |
| C17* | 33 | Female | Married | Junior College | Accountant | 6 months | Daughter | Mo, Ma | Online purchase of health care products, Electronic BP monitor |

Note: An asterisk indicated that the participant's corresponding caregiver was also interviewed

**Appendix 4**

**Table 1.** Example of the coding process

| Category | Subcategories | Examples of codes | Examples of meaning units | Examples of quotations |
| --- | --- | --- | --- | --- |
| Service Experience | Positive experience | Perceived usefulness | Save time and effort. Select online specialists. Record daily activities. Remind to take medicine. Understand physical condition; | *Because online registration does not require running back and forth, it offers the most convenient option. I can make an appointment to see any specialist I want. For example, every time I make an appointment with a chief physician, he will know my condition, which has many benefits.* |
|  |  | Perceived ease of use | Easy to operate. Operate independently | *It is so simple. Just put your arm in and press it. Before, it was inconvenient for us older adults to put on and take off our clothes, which caused our arms to ache.* |
|  |  | Promoting health management of older adults | Enjoy the convenience of technology. Satisfy one's interests. Adapt to social changes. | *I retired and now live at home. Out of curiosity, I bought a treadmill and use it at home regularly. It feels convenient and refreshing, as it saves me the trouble of going out for a run.* |
|  |  | Gain psychological comfort | Understand changes in one's physical health and reduce feelings of panic. | *When you feel anxious or dizzy, you can measure your heart rate and blood oxygen level. This is very helpful. At least your mind will not be so panicked anymore.* |

**Table 2.** Encoding result

| **Main themes** | **Sub-themes** | **Code** |
| --- | --- | --- |
| Usage and Preference of services | Usage situation | / |
|  | Preferred functions | Online learning or shopping |
|  |  | Real-time health monitoring |
|  |  | Health Management |
|  |  | Remote diagnosis/treatment |
|  |  | Community communication |
| Specific service experiences | Positive experience | Perceived Usefulness |
|  |  | Perceived Ease of Use |
|  |  | Promote health management for older adults |
|  |  | Recover psychological comfort |
|  | Negative experience | Low credibility |
|  |  | Complex operation |
|  |  | Increase physical and mental burden. |
| Influencing factors | Promoting factors | Health needs |
|  |  | Excellent user experience |
|  |  | Subjective norms |
|  |  | Government support |
|  | Obstructive factors | Low level of digital health literacy |
|  |  | Weak economic foundation |
|  |  | Lack of awareness of some digital health services |
|  |  | Low service security |
|  |  | Little free time |
| Suggestions and Expectations | Improve the age-friendly and personalised design of the services | / |
|  | Strengthen the intensity of supervision and review | / |
|  | Offer on-site assistance | / |
|  | Encourage digital feedback and peer support | / |
|  | Establish an authoritative platform | / |
|  | Increase the level of publicity | / |
